# Tenofovir Alafenamide Versus Entecavir as Switch Therapy After Tenofovir Disoproxil Fumarate in Patients with Chronic Hepatitis B

**DOI:** 10.3390/jcm15166257

**Published:** 2026-08-13

**Authors:** Hsin-Ju Tsai, Cheng-Hao Wu, Po-Yueh Chen, Chia-Chang Chen, Ying-Cheng Lin, Shou-Wu Lee, Yu-Sheng Lin, Yen-Chun Peng, Teng-Yu Lee

**Affiliations:** 1 Division of Gastroenterology and Hepatology, Department of Internal Medicine, Taichung Veterans General Hospital, No. 1650, Sec. 4, Taiwan Boulevard, Taichung 407219, Taiwan; tsaihj@vghtc.gov.tw (H.-J.T.);; 2 Department of Post-Baccalaureate Medicine, College of Medicine, National Chung Hsing University, Taichung 402202, Taiwan; 3 Division of Gastroenterology and Hepatology, Department of Internal Medicine, Ditmanson Medical Foundation Chia-Yi Christian Hospital, Chia-Yi 600566, Taiwan; 4 Department of Sport, Leisure and Health Management, Tainan University of Technology, Tainan City 710302, Taiwan; 5 School of Medicine, Chung Shan Medical University, Taichung 40201, Taiwan; 6 School of Medicine, National Yang Ming Chiao Tung University, Taipei 112304, Taiwan

**Keywords:** chronic hepatitis B, tenofovir alafenamide, entecavir, treatment switch, renal safety

## Abstract

**Background**: Tenofovir alafenamide (TAF) and entecavir (ETV) are recommended alternatives to tenofovir disoproxil fumarate (TDF) for patients with chronic hepatitis B (CHB) at increased risk of kidney injury. However, data from direct head-to-head comparisons of their long-term renal safety and antiviral efficacy after switching from TDF are lacking. We aimed to compare these outcomes between TAF and ETV. **Methods**: This multicenter retrospective cohort study included consecutive CHB patients who switched from TDF to either TAF or ETV between January 2012 and December 2021. Inverse probability of treatment weighting using the propensity score was applied to balance the baseline characteristics. Changes in estimated glomerular filtration rate (eGFR) were assessed using a linear mixed-effects model. Renal dysfunction was defined as a decline of at least one GFR category. **Results**: A total of 235 patients were included (TAF, *n* = 168; ETV, *n* = 67). After adjustment for key risk factors, the mean eGFR decline over 36 months was not significantly different between the TAF and ETV groups (−3.21 mL/min/1.73 m^2^ [95% CI, −4.98 to −1.44] vs. −3.49 mL/min/1.73 m^2^ [95% CI, −9.49 to −2.50]; *p* = 0.856). The 3-year cumulative incidence of renal dysfunction was also not significantly different between groups (16.6% [95% CI, 10.5–23.3] vs. 7.8% [95% CI, 2.7–16.2]; *p* = 0.135). Alanine aminotransferase normalization rates (83.0% vs. 78.8%; *p* = 0.575) and virological suppression rates (97.8% vs. 97.1%; *p* = 1.000) were similarly high in both groups. **Conclusions**: TAF and ETV demonstrated similar long-term renal safety and antiviral efficacy, supporting both agents as reasonable switch options for CHB patients requiring TDF discontinuation because of renal safety concerns.

## 1. Introduction

Chronic hepatitis B virus (HBV) infection remains a major cause of chronic liver disease worldwide [1]. Although nucleos(t)ide analogue (NA) therapy can effectively inhibit HBV replication, complete HBV eradication remains unachievable. Therefore, in patients with chronic hepatitis B (CHB), long-term NA therapy is recommended by current international guidelines to prevent adverse liver-related outcomes, including cirrhosis and hepatocellular carcinoma (HCC). Among available agents, tenofovir disoproxil fumarate (TDF) is recommended as a first-line therapy because of its potent antiviral activity and high genetic barrier to resistance [2,3,4]. However, concerns regarding long-term renal safety have increasingly emerged, particularly with prolonged exposure [5,6,7,8,9].

The nephrotoxicity associated with TDF has been attributed to elevated plasma tenofovir exposure [10,11]. In contrast, tenofovir alafenamide (TAF), a novel prodrug of tenofovir, delivers the active metabolite more efficiently into hepatocytes while reducing plasma tenofovir concentrations by approximately 89%, thereby potentially reducing the risk of nephrotoxicity [12,13,14]. A phase 3 randomized trial evaluating patients switched from TDF to TAF demonstrated significant improvement in estimated glomerular filtration rate (eGFR) at Week 48 without compromising antiviral efficacy [15]. In East Asian subgroup analyses, the decline in eGFR observed during TDF therapy partially reversed after switching to TAF [16,17]. Furthermore, real-world studies have consistently reported stable or improved renal function after switching from TDF to TAF, including among patients with chronic kidney disease (CKD) [18,19,20,21,22].

Entecavir (ETV) is a recommended alternative for CHB patients at increased risk of kidney injury. It has traditionally been considered renally safe, with a lower incidence of renal dysfunction than TDF in previous studies [5,6,7,8,9]. Studies involving patients who developed renal dysfunction during TDF therapy have also shown improvements in both glomerular and tubular renal markers after switching to ETV [23]. Accordingly, current international guidelines recommend switching from TDF to either TAF or ETV in patients at risk for kidney injury, such as older adults and those with pre-existing renal impairment [2,24]. This issue is particularly relevant in aging CHB populations, in whom renal comorbidities are increasingly prevalent.

Despite these findings, the optimal switch strategy after TDF discontinuation remains unclear. Recent retrospective studies comparing CHB patients treated with TAF or ETV have yielded inconsistent findings, with some reporting a greater risk of renal decline with ETV, whereas others found no significant difference [9,25,26]. Importantly, these studies did not specifically focus on patients switched from TDF. To date, no head-to-head study has directly compared the long-term renal outcomes of TAF and ETV in this clinical setting. Clinically, an optimal switch strategy should preserve not only renal safety but also sustained antiviral efficacy. Therefore, this multicenter cohort study aimed to compare the long-term renal safety and antiviral efficacy of switching from TDF to either TAF or ETV in patients with CHB.

## 2. Patients and Methods

### 2.1. Study Design and Subjects

This retrospective multicenter cohort study was conducted at Taichung Veterans General Hospital (TCVGH) and Ditmanson Medical Foundation Chia-Yi Christian Hospital. Consecutive patients with CHB who received TDF and subsequently switched to either TAF or ETV between 1 January 2012 and 31 December 2021 were included. Clinical outcomes were assessed through 31 December 2024 by reviewing electronic medical records. The study was conducted according to the guidelines of the Declaration of Helsinki and approved by the Institutional Review Board/Ethics Committee of Taichung Veterans General Hospital (protocol code [CE23094C-2] and date of approval [20 February 2025]) and Ditmanson Medical Foundation Chia-Yi Christian Hospital (protocol code [IRB2021044] and date of approval [19 May 2025]). Patient consent was waived due to the retrospective nature of the study.

The patient selection process is illustrated in Supplementary Figure S1. The inclusion criteria were as follows: (1) age ≥ 18 years, (2) persistent HBsAg positivity for at least 6 months, and (3) prior TDF monotherapy followed by a switch to either TAF or ETV monotherapy. The exclusion criteria were: (1) TDF therapy duration of less than 6 months, (2) TAF or ETV therapy duration of less than 12 months, (3) end-stage renal disease (eGFR < 15 mL/min/1.73 m^2^), (4) decompensated liver cirrhosis, defined as the presence of refractory ascites or hepatic encephalopathy, (5) co-infection with the hepatitis C virus or human immunodeficiency virus, (6) documented NA-resistant HBV infection, (7) HCC diagnosed within 6 months after switching to TAF or ETV, (8) uncontrolled malignancies, and (9) incomplete or missing clinical data. Patients with HCC diagnosed within 6 months after treatment switching were excluded to minimize the potential influence of pre-existing occult HCC on treatment selection and subsequent outcomes.

### 2.2. Study Outcomes

The primary outcome was renal safety after switching to TAF or ETV, assessed by longitudinal changes in eGFR and the cumulative incidence of renal dysfunction. eGFR was calculated using the 2021 Chronic Kidney Disease Epidemiology Collaboration (CKD-EPI) creatinine equation [27]. Baseline eGFR was determined within 1 month before treatment switching, and subsequent changes were evaluated every 3 months after switching. Renal dysfunction was defined as a decline of at least one GFR category from baseline during follow-up (e.g., from Grade 2 to 3a or from 3a to 3b). GFR categories were classified as follows: normal renal function (Grade 1, eGFR ≥ 90 mL/min/1.73 m^2^), mildly decreased renal function (Grade 2, eGFR 60–89 mL/min/1.73 m^2^), mild to moderate renal impairment (Grade 3a, eGFR 45–59 mL/min/1.73 m^2^), moderate to severe renal impairment (Grade 3b, eGFR 30–44 mL/min/1.73 m^2^), and severe renal impairment (Grade 4, eGFR 15–29 mL/min/1.73 m^2^) [28].

The secondary outcome was antiviral efficacy after switching to TAF or ETV, assessed by alanine aminotransferase (ALT) normalization and virological suppression rates. Serum ALT and HBV DNA levels were measured approximately every 3 and 6 months, respectively, after treatment switching. ALT normalization was defined as ≤35 U/L for men and ≤25 U/L for women [3]. The lower limit of quantification for serum HBV DNA was 10 IU/mL. Risk factors associated with lack of ALT normalization and persistent viremia were also analyzed. Lack of ALT normalization was defined as ALT levels above the upper limit of normal in three consecutive follow-up tests after switching. Persistent viremia was defined as detectable HBV DNA in two consecutive follow-up measurements despite ongoing therapy.

Exploratory liver-related outcomes included the development of HCC and hepatic decompensation during follow-up.

### 2.3. Risk Factor Assessment

CKD was defined according to Kidney Disease: Improving Global Outcomes (KDIGO) criteria as abnormalities in renal structure or function persisting for more than 3 months [28]. These abnormalities included a GFR < 60 mL/min/1.73 m^2^, proteinuria, or hematuria. Diabetes was defined as a fasting plasma glucose level ≥ 126 mg/dL, glycosylated hemoglobin (HbA1c) ≥ 6.5%, or the use of antidiabetic medications. Hypertension was defined as systolic blood pressure ≥ 130 mmHg, diastolic blood pressure ≥ 85 mmHg, or the use of antihypertensive medications. Metabolic dysfunction-associated steatotic liver disease (MASLD) was defined as hepatic steatosis detected on imaging, along with at least one metabolic risk factor, including diabetes, hypertension, dyslipidemia or a body mass index (BMI) > 23 kg/m^2^ [29]. Hyperuricemia was defined as a serum uric acid level > 7.0 mg/dL for men and >6.0 mg/dL for women, or treatment for hyperuricemia [30]. Information on exposure to potentially nephrotoxic medications after treatment switching, such as nonsteroidal anti-inflammatory drugs (NSAIDs), was extracted from medical records.

In accordance with clinical guidelines, indications for switching from TDF to TAF or ETV, including age > 60 years, osteoporosis, and renal impairment, were extracted from medical records [24]. Osteoporosis was defined as a T-score ≤ −2.5 on dual-energy X-ray absorptiometry of the lumbar spine, femoral neck, or total hip. Renal impairment was defined as eGFR < 60 mL/min/1.73 m^2^, albuminuria, or hypophosphatemia. Albuminuria was defined as a urine albumin-to-creatinine ratio > 30 mg/g or moderate dipstick proteinuria in two consecutive urine samples. Hypophosphatemia was defined as a serum phosphate level < 2.5 mg/dL.

### 2.4. Statistical Analysis

Continuous variables are presented as medians with interquartile ranges (IQRs), whereas categorical variables are presented as counts and percentages. Continuous variables were compared using the Mann–Whitney U test, while categorical variables were compared using the chi-square test or Fisher’s exact test, as appropriate. Inverse probability of treatment weighting (IPTW) using the propensity score was applied to balance the baseline characteristics between the treatment groups. Propensity scores consisted of age, gender, baseline eGFR, chronic kidney disease, HBeAg status, duration of prior TDF, cirrhosis, diabetes, and hypertension. Covariate balance before and after weighting was assessed using standardized mean differences (SMDs), with an absolute SMD < 0.1 considered indicative of adequate balance. To evaluate longitudinal changes in renal function after switching from TDF to TAF or ETV, repeated eGFR measurements were analyzed using an IPTW-weighted linear mixed-effects model with patient-specific random intercepts to account for within-subject correlation over time. Treatment group, time, and group-by-time interaction were included as fixed effects. The annual rate of eGFR change was expressed as the estimated slope coefficient (mL/min/1.73 m^2^/year). The cumulative incidences of renal dysfunction, HCC, and hepatic decompensation were estimated using the Kaplan–Meier method and compared between groups using the log-rank test. Cox proportional hazards regression models were used to identify factors associated with renal dysfunction, with results reported as hazard ratios (HRs) and 95% confidence intervals (CIs). The proportions of patients achieving ALT normalization and undetectable serum HBV DNA were compared using the chi-square test. Logistic regression analysis was performed to identify factors associated with lack of ALT normalization and persistent viremia, with results reported as odds ratios (ORs) and 95% CIs. Variables with *p* < 0.05 in univariate analyses were included in all subsequent multivariable models. This approach was adopted to reduce the risk of model overfitting, particularly for analyses with a limited number of outcome events. All statistical tests were two-tailed, and a *p* value < 0.05 was considered statistically significant. All statistical analyses were performed using IBM SPSS Statistics for Windows, version 24.0 (IBM Corp., Armonk, NY, USA) and R version 4.5.1 (R Foundation for Statistical Computing, Vienna, Austria).

## 3. Results

### 3.1. Baseline Characteristics

A total of 235 eligible patients were included in the final analysis, including 168 in the TAF group and 67 in the ETV group (Supplementary Figure S1). Baseline characteristics were generally balanced between groups (Table 1). Median baseline eGFR was 87.7 mL/min/1.73 m^2^ in the TAF group and 88.0 mL/min/1.73 m^2^ in the ETV group, while pre-existing CKD was present in 17.3% and 14.9% of patients, respectively. Approximately one-fourth of patients in both groups had compensated cirrhosis, and over 80% had serum HBV DNA levels below 10 IU/mL at treatment switching. HBeAg positivity was more frequent in the TAF group than in the ETV group (24.2% vs. 10.4%). Patients who switched to TAF had a longer duration of prior TDF treatment than those who switched to ETV. Regarding the reasons for switching from TDF, approximately one-third of patients in both groups switched because of age > 60 years or concerns regarding long-term TDF-related adverse effects. Switching due to osteoporosis (12.5% vs. 10.4%) or renal impairment (19.0% vs. 20.9%) was similar between groups. However, after being adjusted by IPTW, the baseline covariates were well-balanced between the two study groups.

### 3.2. Changes in Renal Function

As shown in Table 2, the adjusted annual rate of eGFR decline after treatment switching was not statistically significant between the two groups (TAF vs. ETV: −0.23 mL/min/1.73 m^2^/year [95% CI, −3.03 to 2.56]; *p* = 0.869). Factors significantly associated with faster eGFR decline in adjusted analysis included older age and lower baseline eGFR.

**Figure 1:**
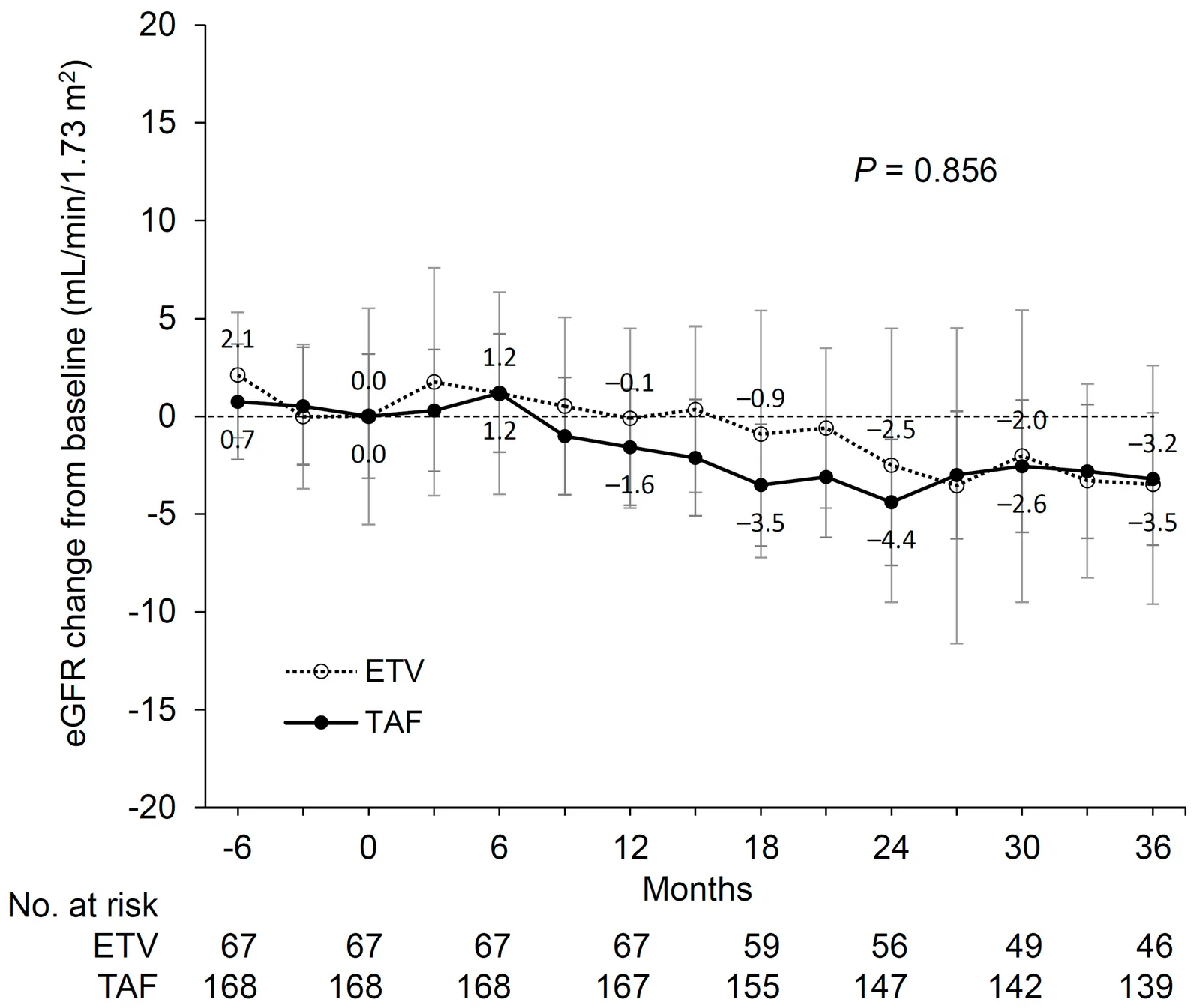
Adjusted mean change in eGFR from baseline with 95% confidence intervals, estimated using linear mixed-effects model treating follow-up visit as a categorical variable. Points represent adjusted mean changes in eGFR, and vertical error bars represent 95% confidence intervals. eGFR, estimated glomerular filtration rate; ETV, entecavir; TAF, tenofovir alafenamide.

Adjusted mean change in eGFR after switching treatment was shown in Supplementary Figure S2. The estimated 36-month changes in eGFR were −3.21 mL/min/1.73 m^2^ (95% CI, −4.98 to −1.44) in the TAF group and −3.49 mL/min/1.73 m^2^ (95% CI, −9.49 to 2.50) in the ETV group, with a between-group difference of −0.64 mL/min/1.73 m^2^ (95% CI, −7.62 to 6.33; *p* = 0.856) (Figure 1). Following treatment switching, patients in both groups showed a slight increase in adjusted mean eGFR from baseline at 6 months (TAF vs. ETV, 1.19 mL/min/1.73 m^2^ [95% CI, 0.05 to 2.33] vs. 1.18 mL/min/1.73 m^2^ [95% CI, −1.10 to 3.46]; *p* = 0.867). Thereafter, both groups showed gradual declines in adjusted mean eGFR over time.

### 3.3. Sensitivity Analysis Using the Common-Era Cohort

Before October 2018, patients could only switch from TDF to ETV because TAF was not yet available at either participating hospital. To minimize potential calendar-time bias, we performed a sensitivity analysis restricted to the common-era cohort, during which both TAF and ETV were available at both participating hospitals. The cohort included 206 patients (TAF, *n* = 168; ETV, *n* = 38). Baseline renal function was similar between the two groups, with median eGFR values of 87.7 and 85.0 mL/min/1.73 m^2^ in the TAF and ETV groups, respectively (*p* = 0.864). The linear mixed-effects analysis showed no significant difference in the annual eGFR slope between the two groups (slope difference, −0.20 mL/min/1.73 m^2^/year [95% CI, −2.22 to 1.82]; *p* = 0.401). Similarly, the estimated between-group difference in eGFR change over 36 months was not significant (−0.59 mL/min/1.73 m^2^ [95% CI, −6.65 to 5.46]; *p* = 0.848) (Supplementary Figure S3). These results were consistent with the primary analysis, suggesting that calendar-time bias did not materially influence the study findings.

### 3.4. Incidence of Renal Dysfunction

**Figure 2:**
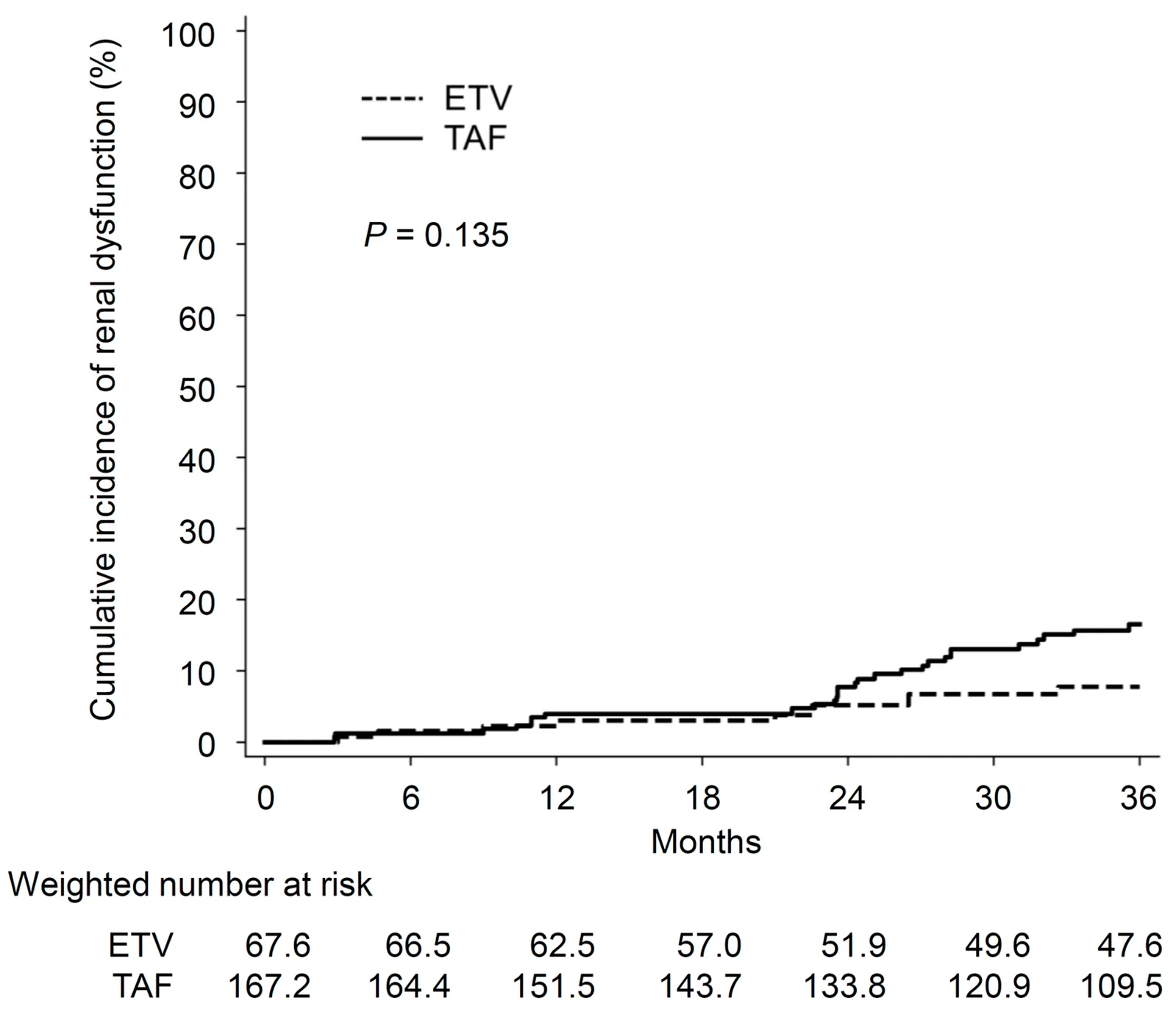
Cumulative incidence of renal dysfunction, defined as a decline of at least one GFR category from baseline. ETV, entecavir; TAF, tenofovir alafenamide.

After IPTW, the cumulative incidence of renal dysfunction at 3 years did not differ significantly between groups (TAF: 16.6% [95% CI, 10.5–23.3] vs. ETV: 7.8% [95% CI, 2.7–16.2]; *p* = 0.135) (Figure 2). A total of 24 patients in the TAF group and 8 in the ETV group developed renal dysfunction. All experienced a one-grade decline in GFR category, with no progression to end-stage renal disease or requirement for hemodialysis.

Univariable Cox regression identified older age, lower baseline eGFR, pre-existing CKD, and cirrhosis as factors significantly associated with renal dysfunction. Because baseline eGFR and pre-existing CKD were highly correlated, they were entered into separate multivariable models to avoid multicollinearity. In multivariable Cox regression Model 1, after adjusting for age and cirrhosis, lower baseline eGFR remained an independent predictor of renal dysfunction (adjusted HR 1.03 [95% CI, 1.00–1.05]; *p* = 0.016) (Table 3). No significant difference in renal dysfunction risk was observed between the TAF and ETV groups (adjusted HR 0.90 [95% CI, 0.39–2.03]; *p* = 0.792). In Model 2, after replacing baseline eGFR with pre-existing CKD while adjusting for age and cirrhosis, both older age (adjusted HR 1.04 [95% CI, 1.01–1.07]; *p* = 0.011) and pre-existing CKD (adjusted HR 2.97 [95% CI, 1.26–7.00]; *p* = 0.013) remained independently associated with renal dysfunction. As a sensitivity analysis, renal dysfunction was alternatively defined as a ≥30% decline in eGFR from baseline. At 3 years, the cumulative incidence was 8.8% (95% CI, 4.0–14.1) in the TAF group and 3.9% (95% CI, 0.7–9.5) in the ETV group (*p* = 0.276) (Supplementary Figure S4). Consistent with the primary analysis, no significant difference was observed between the two groups (TAF vs. ETV, adjusted HR 2.14 [95% CI, 0.62–7.47], *p* = 0.231).

### 3.5. ALT Normalization and Virological Suppression Evaluations

**Figure 3:**
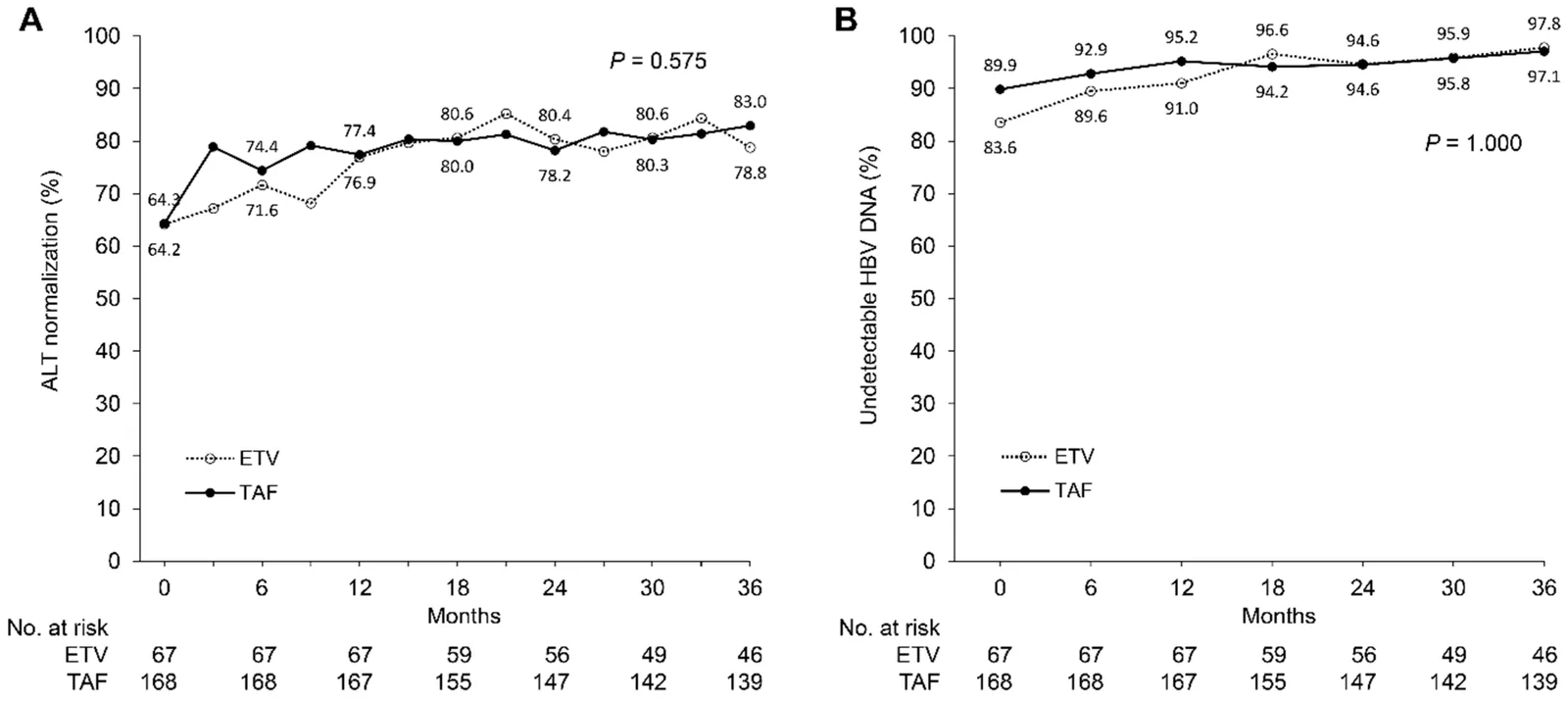
Proportions of patients achieving (**A**) ALT normalization and (**B**) virological suppression with undetectable serum HBV DNA during follow-up. ALT, alanine aminotransferase; DNA, deoxyribonucleic acid; ETV, entecavir; HBV, hepatitis B virus; TAF, tenofovir alafenamide.

Baseline ALT normalization rates were similar between groups (approximately 64% in both groups; *p* = 0.988). ALT normalization improved after treatment switching and plateaued after 18 months, with no significant between-group difference at 36 months (TAF vs. ETV: 83.0% vs. 78.8%; *p* = 0.575) (Figure 3A). In multivariable logistic regression analysis, baseline detectable serum HBV DNA (OR 2.72 [95% CI, 1.16–6.35]; *p* = 0.021) and coexisting MASLD (OR 2.09 [95% CI, 1.05–4.15]; *p* = 0.036) were independently associated with lack of ALT normalization. Virological suppression improved in both groups after treatment switching, with no significant between-group difference at 36 months (TAF vs. ETV: 97.8% vs. 97.1%; *p* = 1.000) (Figure 3B). Baseline HBeAg positivity was the only independent predictor of persistent viremia (OR 14.48 [95% CI, 3.47–60.3]; *p* < 0.001).

### 3.6. Exploratory Clinical Outcomes

The cumulative incidences of HCC and hepatic decompensation at 3 years did not differ significantly between groups. The 3-year cumulative incidence of HCC was 6.3% in the TAF group and 4.0% in the ETV group (*p* = 0.546), while the corresponding incidence of hepatic decompensation was 1.4% and 2.0%, respectively (*p* = 0.784) (Supplementary Figure S5). No cases of end-stage renal disease, dialysis, renal transplantation, liver transplantation, or death occurred during follow-up.

## 4. Discussion

This multicenter cohort study provides one of the first direct comparisons of the long-term renal safety and antiviral efficacy of switching from TDF to either TAF or ETV in patients with chronic hepatitis B. Our results showed that both switch strategies were associated with similar long-term renal outcomes, including eGFR trajectories and cumulative incidences of renal dysfunction over 36 months. In addition, antiviral efficacy remained well preserved in both groups, with no significant differences in ALT normalization or virological suppression. These findings suggest that both TAF and ETV can be considered potential switch options for CHB patients requiring discontinuation of TDF, particularly those at increased risk of kidney injury.

The primary mechanism of TDF-induced renal injury involves tenofovir accumulation in proximal renal tubular cells, leading to tubular dysfunction and nephrotoxicity. In contrast, TAF reduces systemic tenofovir exposure through improved plasma stability and more efficient intracellular delivery of the active metabolite at a lower dose [13,14]. Unlike TDF, ETV is not associated with tenofovir-related tubular toxicity, providing a mechanistic rationale for the use of both TAF and ETV as switch options in patients with renal safety concerns. In our cohort, eGFR slightly improved within 6 months after switching to TAF, consistent with previous studies suggesting partial reversibility of TDF-related renal injury after treatment modification [15,18,19,20,21,22]. This early improvement supports the notion that discontinuation of TDF may halt ongoing tubular injury and allow partial renal recovery. Thereafter, eGFR declined gradually at similar rates in both groups, suggesting that long-term renal trajectories are driven more by underlying patient factors than by the choice of antiviral agent [31]. Older age, lower baseline eGFR, and pre-existing CKD were independent predictors of renal dysfunction, underscoring the need for continued renal monitoring after treatment switching, particularly in high-risk patients.

Several previous studies reported stabilization or improvement in renal function after switching from TDF, whereas both treatment groups in our cohort exhibited modest declines in eGFR over time [15,23]. This difference is likely attributable to variations in study populations and follow-up duration. Previous studies either evaluated only short-term renal outcomes or selectively enrolled patients with TDF-associated nephrotoxicity, who had greater potential for renal recovery after TDF withdrawal. In contrast, our real-world cohort included patients with generally preserved baseline renal function and broader clinical indications for switching. Importantly, neither treatment group showed evidence of accelerated renal decline after switching. Rather, the modest annual decline in eGFR was consistent with the expected effects of physiological aging and chronic comorbidities [32,33], suggesting that discontinuation of TDF may attenuate additional nephrotoxicity without completely preventing the background decline in renal function. These findings support continued renal monitoring after switching to either TAF or ETV.

Antiviral efficacy remained excellent in both groups after switching from TDF, with similarly high rates of long-term virological suppression in patients receiving either TAF or ETV. These findings suggest that both high-potency NAs can effectively maintain viral suppression after treatment modification. ALT normalization also improved progressively after switching and plateaued at approximately 80% in both groups, with no meaningful difference between treatment arms. Notably, the choice of antiviral agent appeared to have less influence on ALT normalization than underlying metabolic comorbidities. In particular, coexisting MASLD was independently associated with persistent ALT elevation despite virological suppression. This finding highlights that persistent ALT abnormalities during NA therapy should not be attributed solely to HBV activity and should prompt evaluation for coexisting metabolic liver disease and other non-viral causes of liver inflammation in patients with CHB [34].

Unlike previous switch studies reporting stable virological suppression after treatment switching [15,23], the proportion of patients with undetectable HBV DNA increased during follow-up in both treatment groups. This difference is likely explained by differences in the study populations. Previous studies largely enrolled patients with complete virological suppression before switching, whereas our real-world cohort included patients with ongoing viral replication, many of whom were HBeAg-positive and had received a shorter duration of prior TDF therapy [35]. The improvement in virological suppression among prior TDF users may reflect continued or additional viral suppression after switching therapy; however, the mechanisms underlying this additional effect, such as improved adherence or increased drug exposure, require further investigation.

Previous LAM exposure deserves particular consideration when switching from TDF to ETV. Although patients with documented NA resistance were excluded, archived LAM-resistant variants may persist in intrahepatic covalently closed circular DNA despite undetectable serum HBV DNA and are not routinely detectable in clinical practice. Viganò et al. reported an increased risk of recurrent viremia after switching to ETV among patients with prior lamivudine exposure [23]. In our cohort, prior LAM exposure was uncommon and balanced between treatment groups, and no clinical evidence suggestive of emergent entecavir resistance was observed during follow-up. Furthermore, prior LAM exposure was not associated with persistent viremia or failure to achieve ALT normalization. Archived resistance mutations cannot be excluded. Caution should therefore be exercised when considering ETV in patients with previous LAM exposure. Even in the absence of documented LAM resistance, avoidance of ETV or close virological monitoring should be considered.

Previous studies comparing renal outcomes between TAF and ETV in patients with CHB have reported inconsistent results, with some suggesting greater renal decline with ETV and others showing no significant difference [9,25,26]. Several factors may explain these discrepancies, including differences in study populations, baseline renal function, follow-up duration, and definitions of renal outcomes. Importantly, most prior studies compared patients receiving TAF or ETV as primary therapy rather than specifically evaluating patients switched from TDF. In contrast, our study focused on a clinically relevant population in whom treatment switching was primarily driven by renal safety concerns. The relatively long follow-up duration and serial assessment of renal function further strengthen our findings and provide clinically meaningful real-world evidence regarding switch therapy selection after TDF discontinuation.

This study has several limitations. First, because this was a retrospective observational study, selection bias and confounding by indication could not be fully avoided. In routine clinical practice, the choice between TAF and ETV may be influenced by patient characteristics, such as pre-existing CKD, osteoporosis, and physician preference, which may also explain the unequal sample sizes between groups. Therefore, we applied IPTW to balance baseline characteristics between the two treatment groups to mitigate potential selection bias, and used multivariable longitudinal and regression analyses to adjust for measured confounders. Nevertheless, residual confounding from unmeasured factors cannot be excluded, and prospective studies with predefined treatment-selection criteria are warranted to confirm our findings. Second, specific biomarkers of renal tubular injury, such as beta-2 microglobulin, were unavailable. Therefore, subtle tubular dysfunction may have been underrecognized despite longitudinal assessment of conventional renal parameters. Third, other long-term safety outcomes associated with NA therapy, including bone mineral density and metabolic changes, were not evaluated. Future prospective studies with comprehensive renal, skeletal, and metabolic assessments are warranted to further validate our findings.

In conclusion, switching from TDF to either TAF or ETV was associated with similar long-term renal outcomes and sustained antiviral efficacy in patients with chronic hepatitis B. Both agents may be considered potential switch options for patients requiring TDF discontinuation because of renal safety concerns.

## Figures and Tables

**Table 1:** Baseline characteristics of the study cohort. Continuous variables are expressed in median (interquartile range).

	Tenofovir Alafenamide *n* = 168		Entecavir *n* = 67		SMD Before IPTW	SMD After IPTW
Age, years	51.1	(40.6–65.2)	53.3	(41.5–63.9)	0.031	0.100
Male, *n* (%)	112	(66.7)	48	(71.6)	0.050	0.080
Serum creatinine, mg/dL	0.94	(0.81–1.08)	0.90	(0.78–1.09)	0.116	0.004
eGFR, mL/min/1.73 m^2^	87.7	(71.1–101.0)	88.0	(74.4–101.9)	0.132	0.047
Chronic kidney disease, *n* (%)	29	(17.3)	10	(14.9)	0.023	0.016
Alanine aminotransferase, U/L	26	(20–39)	27	(21–41)	0.069	0.051
Total bilirubin, mg/dL	0.7	(0.5–0.9)	0.8	(0.6–1.1)	0.028	0.091
Platelet, 10^9^/L	208.0	(148.8–258.5)	166.0	(140.5–225.0)	0.052	0.077
Undetectable HBV DNA, *n* (%)	151	(89.9)	56	(83.6)	0.087	0.054
Positive HBeAg, *n* (%)	41	(24.4)	7	(10.4)	0.140	0.093
Prior TDF duration, years	3.9	(2.4–5.9)	3.0	(1.6–3.2)	0.859	0.098
Prior LAM exposure, *n* (%)	7	(4.2)	2	(3.0)	0.064	0.005
Cirrhosis (Child-Pugh A), *n* (%)	43	(25.6)	18	(26.9)	0.013	0.047
Diabetes, *n* (%)	25	(14.9)	7	(10.4)	0.044	0.026
Hypertension, *n* (%)	39	(23.2)	17	(25.4)	0.022	0.006
Dyslipidemia, *n* (%)	31	(18.5)	11	(16.4)	0.054	0.032
Body mass index, kg/m^2^	23.7	(21.3–25.9)	24.7	(22.2–26.9)	0.098	0.088
Coexisting MASLD, *n* (%)	47	(28.0)	15	(22.4)	0.110	0.091
Hyperuricemia, *n* (%)	20	(11.9)	8	(11.9)	0.001	0.046
Concurrent NSAIDs use, *n* (%)	21	(12.5)	7	(10.4)	0.064	0.050
Reason for switching						
Age > 60 years, *n* (%)	61	(36.3)	23	(34.3)	0.042	0.094
Osteoporosis, *n* (%)	21	(12.5)	7	(10.4)	0.064	0.086
Renal impairment, *n* (%) ^a^	32	(19.0)	14	(20.9)	0.046	0.093
eGFR < 60 mL/min/1.73 m^2^, *n* (%)	25	(14.9)	9	(13.4)		
Albuminuria, *n* (%)	8	(4.8)	3	(4.5)		
Hypophosphatemia, *n* (%)	4	(2.4)	2	(3.0)		
Concerning adverse effects, *n* (%)	54	(32.1)	23	(34.3)	0.051	0.071

Abbreviations: DNA, deoxyribonucleic acid; eGFR, estimated glomerular filtration rate; HBeAg, hepatitis B e antigen; HBV, hepatitis B virus; IPTW, inverse probability of treatment weighting; LAM, lamivudine; MASLD, metabolic dysfunction-associated steatotic liver disease; NSAIDs, nonsteroidal anti-inflammatory drugs; SMD, standardized mean difference; TDF, tenofovir disoproxil fumarate. ^a^ Each patient may have more than one situation of renal impairment.

**Table 2:** Adjusted linear mixed-effects analyses of eGFR slope after treatment switching.

	Adjusted Slope Coefficient Difference (95% CI) (mL/min/1.73 m^2^/Year)		*p* Value
TAF vs. ETV	−0.23	(−3.03 to 2.56)	0.869
Age (per 1-year increase)	−0.10	(−0.16 to −0.03)	0.005
Baselines eGFR (per 1 mL/min/1.73 m^2^ decrease)	−0.85	(−0.90 to −0.80)	<0.001
Chronic kidney disease	−1.48	(−3.71 to 0.75)	0.194
HBeAg (positive vs. negative)	−0.40	(−2.05 to 1.25)	0.636
Prior TDF duration (per 1-year increase)	0.60	(−0.05 to 1.25)	0.072
Cirrhosis	0.63	(−0.87 to 2.13)	0.412
Diabetes	1.44	(−0.52 to 3.40)	0.152
Hypertension	0.22	(−1.36 to 1.80)	0.786

Abbreviations: CI, confidence interval; ETV, entecavir; eGFR, estimated glomerular filtration rate; HBeAg, hepatitis B e antigen; TAF, tenofovir alafenamide; TDF, tenofovir disoproxil fumarate.

**Table 3:** Cox proportional hazards analysis of factors associated with renal dysfunction after treatment switching.

	Multivariable Model 1			Multivariable Model 2		
	HR	95% CI	*p* Value	HR	95% CI	*p* Value
TAF vs. ETV	0.90	(0.39–2.03)	0.792	0.86	(0.38–1.95)	0.717
Age, years (per 1-year increase)	1.03	(1.00–1.07)	0.071	1.04	(1.01–1.07)	0.011
Baselines eGFR (per 1 mL/min/1.73 m^2^ decrease)	1.03	(1.00–1.05)	0.016			
Chronic kidney disease				2.97	(1.26–7.00)	0.013
Cirrhosis	1.60	(0.76–3.37)	0.220	1.49	(0.70–3.17)	0.298

Abbreviations: CI, confidence interval; eGFR, estimated glomerular filtration rate; ETV, entecavir; HR, hazard ratio; TAF, tenofovir alafenamide.

## Data Availability

Data are available upon request to the author.

## Supplementary Materials

The following supporting information can be downloaded at: https://www.mdpi.com/article/10.3390/jcm15166257/s1. Figure S1: Flow diagram of study cohort selection; Figure S2: Adjusted mean change in eGFR with 95% confidence intervals in full-cohort; Figure S3: Adjusted mean change in eGFR from baseline with 95% confidence intervals in common-era cohort; Figure S4: The cumulative incidence of a ≥30% decline in eGFR from baseline. At 3 years, the cumulative incidence was 8.8% (95% CI, 4.0–14.1) in the TAF group and 3.9% (95% CI, 0.7–9.5) in the ETV group (P = 0.276); Figure S5: Cumulative incidences of (A) hepatocellular carcinoma and (B) hepatic decompensation.

## Abbreviations

ALT, alanine aminotransferase; BMI, body mass index; CHB, chronic hepatitis B; CKD, chronic kidney disease; CKD-EPI, Chronic Kidney Disease Epidemiology Collaboration; CI, confidence interval; DNA, deoxyribonucleic acid; eGFR, estimated glomerular filtration rate; ETV, entecavir; GFR, glomerular filtration rate; HbA1c, hemoglobin A1c; HBeAg, hepatitis B e antigen; HBV, hepatitis B virus; HCC, hepatocellular carcinoma; HR, hazard ratio; IPTW, inverse probability of treatment weighting; IQR, interquartile range; KDIGO, Kidney Disease: Improving Global Outcomes; LAM, lamivudine; MASLD, metabolic dysfunction-associated steatotic liver disease; NA, nucleos(t)ide analogue; NSAIDs, nonsteroidal anti-inflammatory drugs; OR, odds ratio; SMD, standardized mean difference; TAF, tenofovir alafenamide; TDF, tenofovir disoproxil fumarate.
