# Supplementary material for: Tenofovir Alafenamide Versus Entecavir as Switch Therapy After Tenofovir Disoproxil Fumarate in Patients with Chronic Hepatitis B
Source: J Clin Med. 2026 Aug 13;15(16):6257. doi: 10.3390/jcm15166257 (PMC13513944; doi:10.3390/jcm15166257)

Supplementary material

Supplementary Figure S1. Flow diagram of study cohort selection.

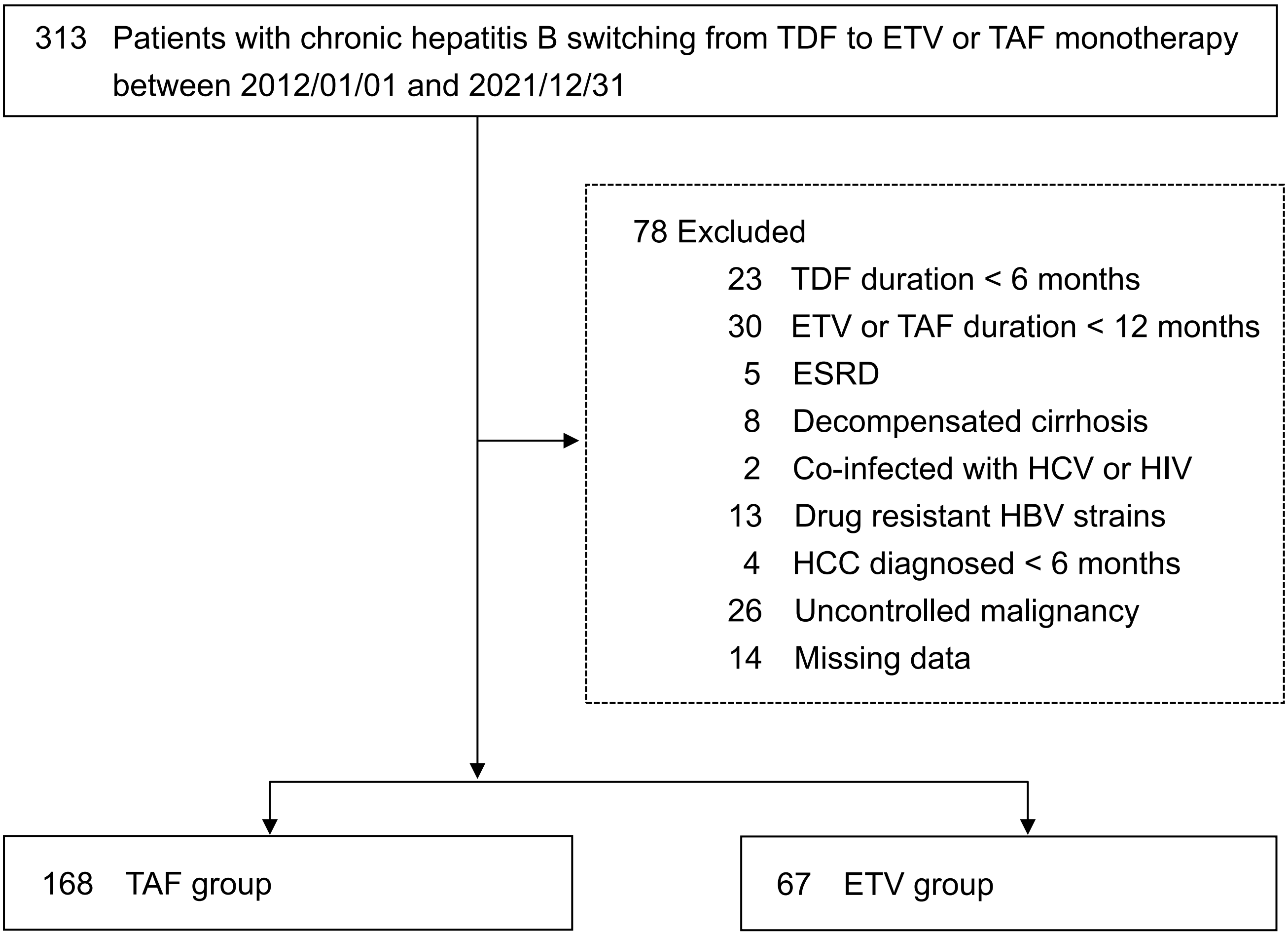

ESRD, end-stage renal disease; ETV, entecavir; HBV, hepatitis B virus; HCC, hepatocellular carcinoma; HCV, hepatitis C virus; HIV, human immunodeficiency virus; TAF, tenofovir alafenamide; TDF, tenofovir disoproxil fumarate.

Supplementary Figure S2. Adjusted mean change in eGFR with 95% confidence intervals in full-cohort. eGFR, estimated glomerular filtration rate; ETV, entecavir; ; TAF, tenofovir alafenamide.

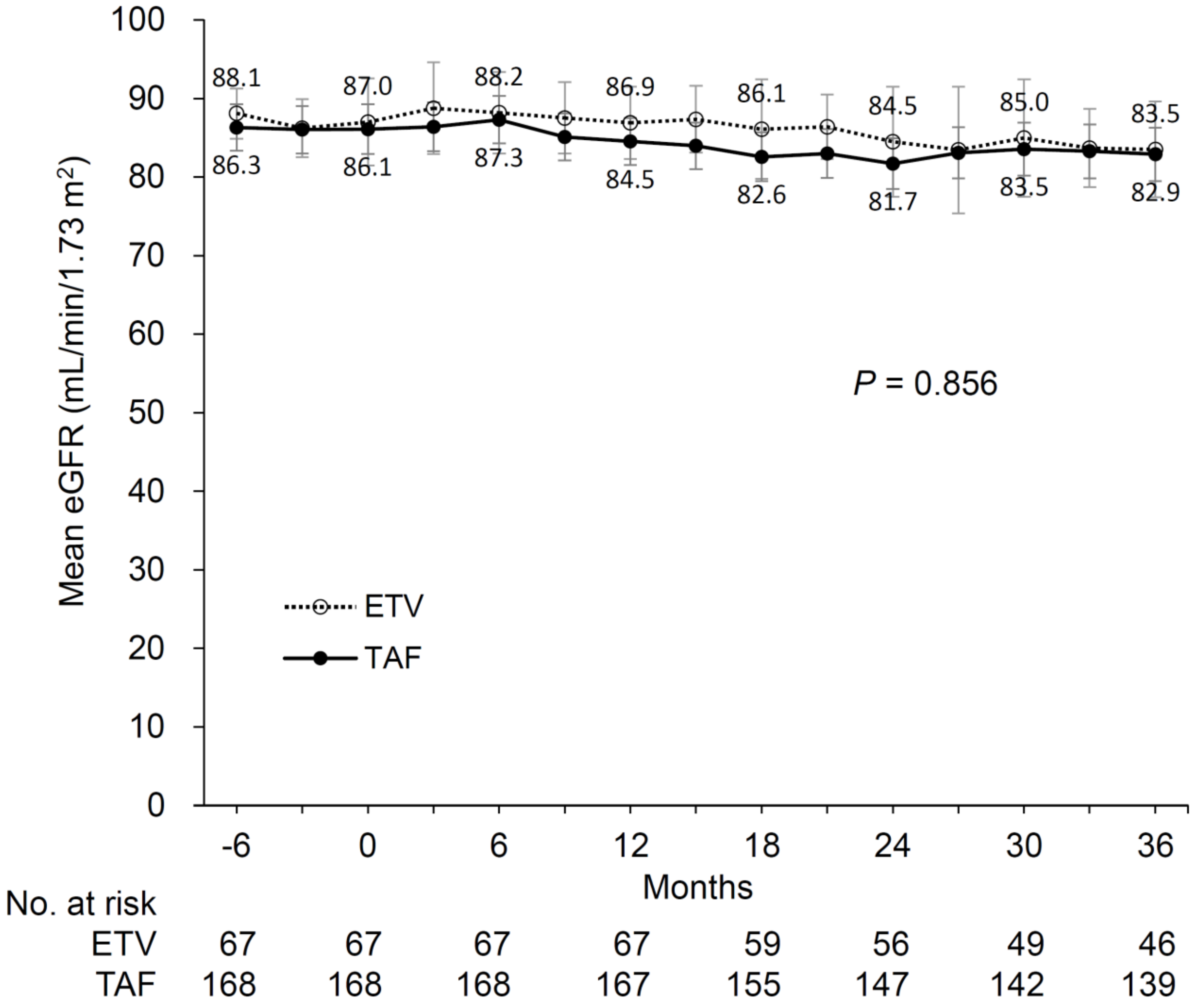

Supplementary Figure S3. Adjusted mean change in eGFR from baseline with 95% confidence intervals in common-era cohort. ETV, entecavir; eGFR, estimated glomerular filtration rate; TAF, tenofovir alafenamide.

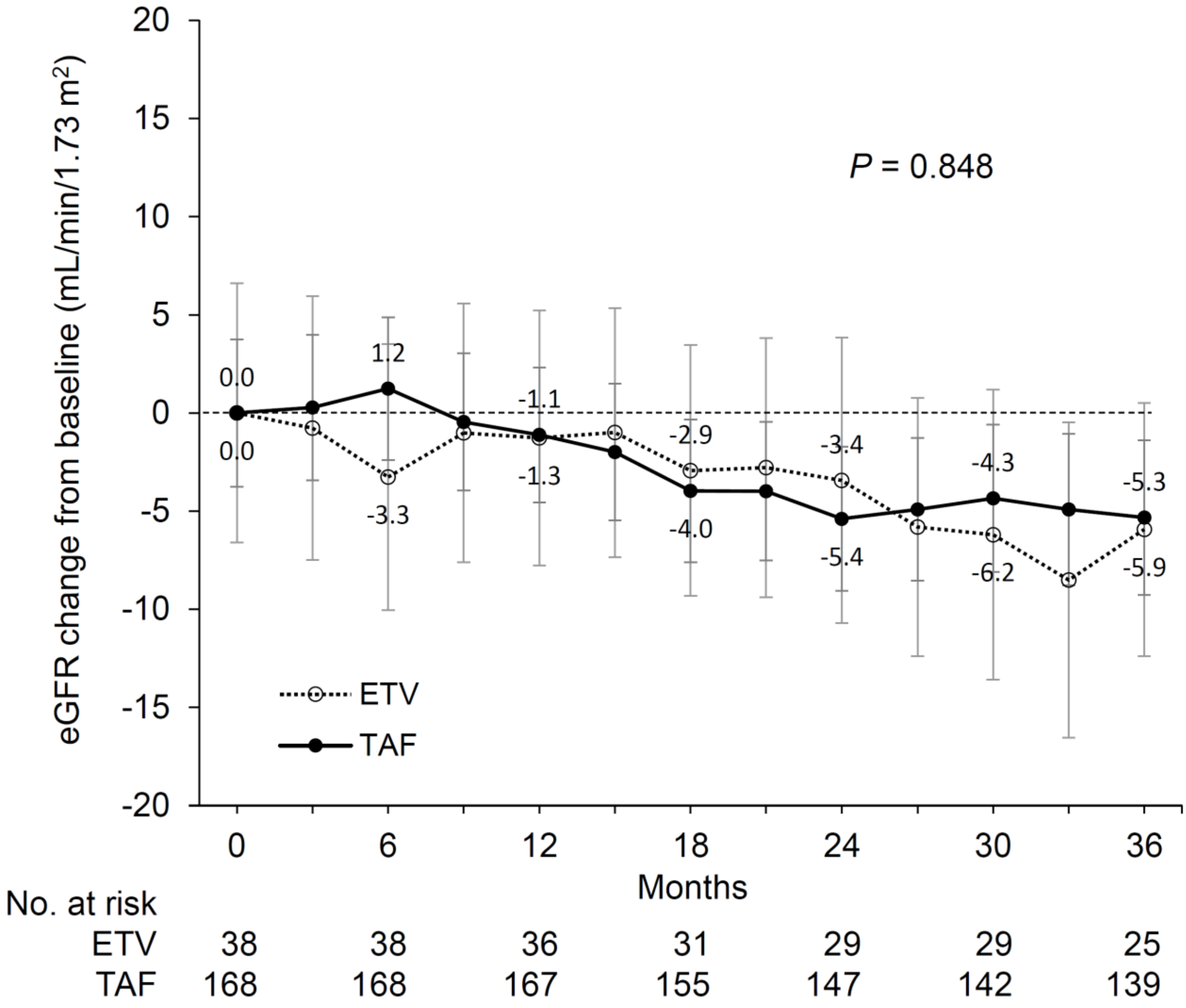

Supplementary Figure S4. The cumulative incidence of a ≥30% decline in eGFR from baseline. At 3 years, the cumulative incidence was 8.8% (95% CI, 4.0–14.1) in the TAF group and 3.9% (95% CI, 0.7–9.5) in the ETV group (*P* = 0.276). eGFR, estimated glomerular filtration rate; ETV, entecavir; TAF, tenofovir alafenamide.

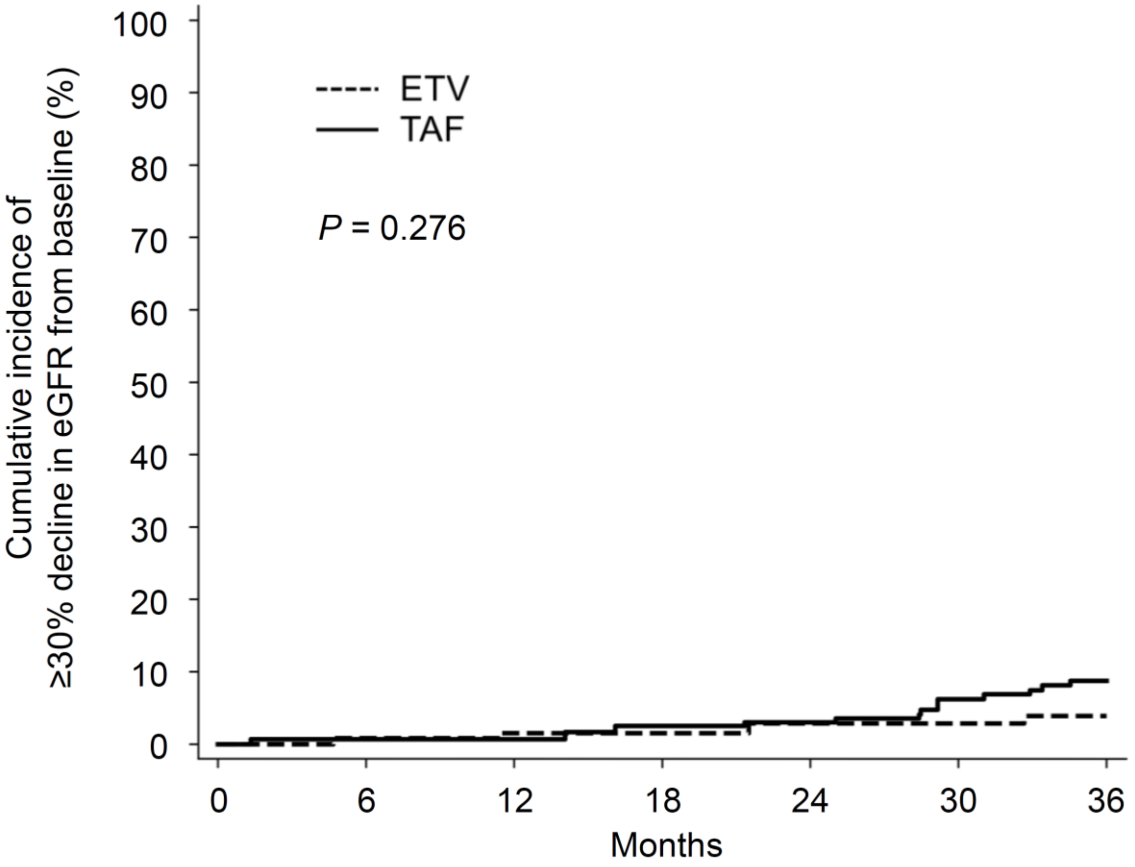

Weighted number at risk

ETV	67.6	67.0	63.0	58.0	52.6	51.1	48.6
TAF	167.2	165.4	155.0	144.3	139.2	129.2	118.5

Supplementary Figure S5. Cumulative incidences of (A) hepatocellular carcinoma and (B) hepatic decompensation. ETV, entecavir; HCC, hepatocellular carcinoma; TAF, tenofovir alafenamide.

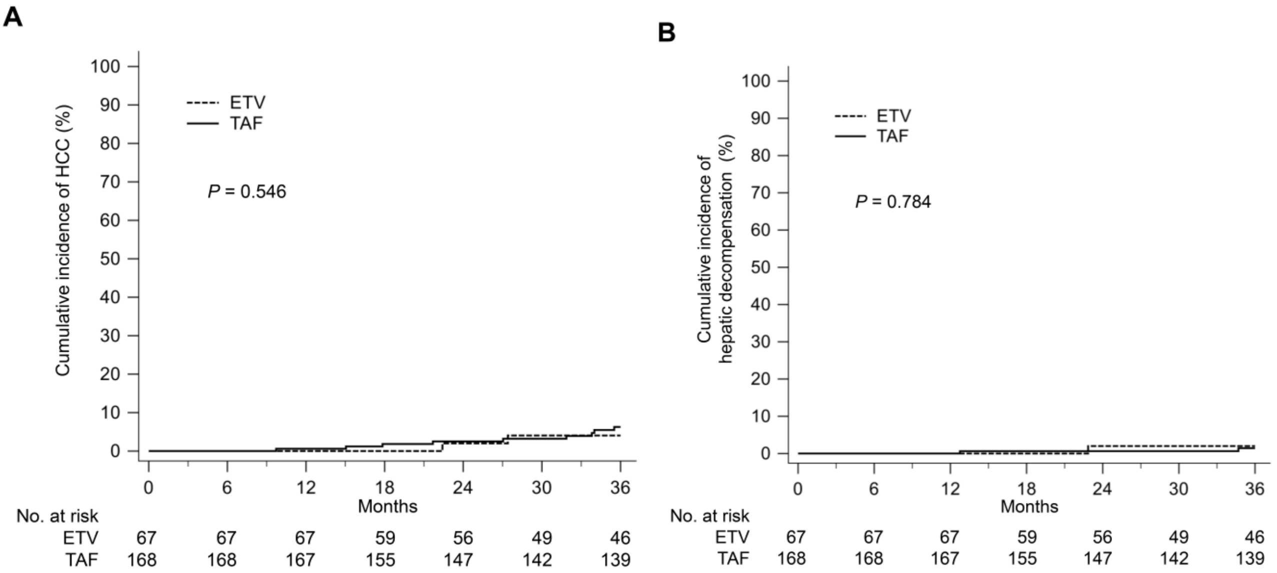

Supplement: Supplementary file 1 [file jcm-15-06257-s001.zip › jcm-4419115-supplementary.pdf]
